# Efficacy of naproxen with or without esomeprazole for pain and inflammation in patients after bilateral third molar extractions: A double blinded crossover study

**DOI:** 10.4317/medoral.21514

**Published:** 2016-12-06

**Authors:** Giovana M. Weckwerth, Luis F. Simoneti, Paulo Zupelari-Gonçalves, Adriana M. Calvo, Daniel T. Brozoski, Thiago J. Dionísio, Elza A. Torres, José-Roberto P. Lauris, Flávio-Augusto C. Faria, Carlos F. Santos

**Affiliations:** 1 DDS, MSc. Department of Biological Sciences, Bauru School of Dentistry, University of São Paulo, Bauru, SP, Brazil; 2 DDS, MSc, PhD, Postdoctoral Fellow. Department of Biological Sciences, Bauru School of Dentistry, University of São Paulo, Bauru, SP, Brazil; 3 MSc, PhD, Laboratory Specialist. Department of Biological Sciences, Bauru School of Dentistry, University of São Paulo, Bauru, SP, Brazil; 4 DDS, MSc, PhD, Professor. Department of Pediatric Dentistry, Orthodontics and Community Health, Bauru School of Dentistry, University of São Paulo, Bauru, SP, Brazil; 5 DDS, MSc, PhD, Associate Professor. Department of Biological Sciences, Bauru School of Dentistry, University of São Paulo, Bauru, SP, Brazil; 6 DDS, MSc, PhD, Professor and Vice-Dean. Department of Biological Sciences, Bauru School of Dentistry, University of São Paulo, Bauru, SP, Brazil

## Abstract

**Background:**

Using a double-blinded randomized crossover design, this study aimed to evaluate acute postoperative pain management, swelling and trismus in 46 volunteers undergoing extractions of the two lower third molars, in similar positions, at two different appointments who consumed a tablet of either NE (naproxen 500 mg + esomepraz ole 20 mg) or only naproxen (500 mg) every 12 hours for 4 days.

**Material and Methods:**

Parameters were analyzed: self-reported pain intensity using a visual analog scale (VAS) pre- and postoperative mouth opening; incidence, type and severity of adverse reactions; total quantity consumed of rescue medication; and pre- and postoperative swelling.

**Results:**

Female volunteers reported significantly more postoperative pain at 1, 1.5, 2, 3 and 4hrs after surgery while also taking their first rescue medication at a time significantly earlier when consuming NE when compared to naproxen (3.7hrs and 6.7hrs). Conversely, no differences were found between each drug group in males.

**Conclusions:**

In conclusion, throughout the entire study, pain was mild after using either drug in both men and women with pain scores on average well below 40mm (VAS), although in women naproxen improved acute postoperative pain management when compared to NE.

**Key words:**Oral surgery, third molar, pain, naproxen, esomeprazole, NSAIDs.

## Introduction

Third molar extraction is the most common surgical procedure in dentistry, and it is commonly used to investigate acute postoperative pain and inflammation (1). In particular, third molar surgery involves the manipulation of the molar and the surrounding soft tissue and bone. The manipulation of the surrounding loose connective mediates the release of inflammatory agents leading to edema, trismus and pain (1,2-4). After third molar extraction, postoperative pain is generally reported to be short term with mild intensity, followed by greater pain intensity 2 to 4 hours after surgery with many patients requiring analgesic medication (4,5). Swelling and trismus are frequent manifestations associated with the inflammatory process originating from oral surgeries (1-4).

Nonsteroidal anti-inflammatory drugs (NSAIDs) have been extensively used to reduce these postoperative complications resulting from third molar surgeries (2-4,6), and are commonly prescribed for the control of chronic and acute inflammatory pain conditions (3,7,8). The therapeutic effects of NSAIDs are mediated by inhibiting cyclooxygenases (COXs). The two isoforms of COXs, COX-1 and COX-2, play important roles in inflammation, pain, and fever via the production of prostaglandins (PGs) and thromboxanes (9). Different types of NSAIDs may non selectively inhibit both COX-1 and COX-2, or selectively inhibit COX-2. In particular, selective NSAIDs that affect COX-1 more so than COX-2 have anti-inflammatory, analgesic and antipyretic effects, but they also often lead to gastrointestinal problems associated with COX-1 inhibition. Moreover, these gastric problems can lead to significant pain independent of the original condition.

It should also be noted that, when compared to men, women have increased pain somatization, sensitivity and intolerance for various types of pain (10). Furthermore, some pain symptoms have been demonstrated to fluctuate with the menstrual cycle (11,12) although further investigations are needed. When examining renal hemodynamic parameters Cherney *et al.* (13) (2008) found that women were significantly sensitive to COX-2 inhibition whereas men were not, and they hypothesized that women might have greater baseline prostaglandin activity when compared to men. They also hypothesized that vasodilatory prostaglandins are more effective in women versus men (13). In animal models, others have implicated estrogen as a mediating factor since estrogen increased vasodilatory prostaglandin synthesis (14-16). Therefore, COX-2 mediated mechanisms may be significantly affected by estrogen and thus by gender.

Naproxen is an NSAID commonly used in dental research to manage postoperative pain and swelling, and several studies have shown that it effectively manages both chronic and acute pain (2,3,8,17). More specifically, 500 mg of oral naproxen has been extensively studied in randomized, double-blind, crossover trials demonstrating its efficacy in managing acute pain, swelling and trismus after bilateral extraction of lower third molars in similar positions (4,6,17,18). However, since this propionic acid derivative is a nonselective inhibitor of COX, it is associated with gastrointestinal problems such as gastric ulcers, gastrointestinal perforations, and stomach bleeding (7) along side its analgesic, antipyretic and anti-inflammatory properties (2,3,7,8,13). In view of these gastrointestinal complications, an enteric coating of esomeprazole magnesium (20 mg) was developed in combination with naproxen (500 mg). In particular, an oral tablet of naproxen/esomeprazole (NE) comes in two dosage strengths, 500/20 mg and 375/20 mg (19).

Briefly, esomeprazole inhibits the H+/K+-ATPase proton pump, and an enteric coating of esomeprazole over naproxen, which is activated by acid should in theory reduce the secretion of protons in the stomach. Namely, naproxen is delayed in its absorption by approximately 26 to 72 minutes while the coating of esomeprazole is absorbed (19). The reduction of stomach acid by esomeprazole in essence may then protect the stomach lining from gastrointestinal problems associated with COX-1 inhibition while the plasma concentration of naproxen increases (19). Therefore, management of acute pain may be delayed with NE compared to naproxen alone, yet gastrointestinal problems might be reduced (19). However it should be noted that local anesthetics typically employed during third molar extraction requiring osteotomies may last approximately 195 minutes after surgery and, thus, the postoperative pain immediately after surgery will be maintained by a combination of local anesthesia and postoperative pain medication (5).

Thus, the aim of this study was to evaluate the clinical efficacy of naproxen (500 mg) versus NE (500/20 mg) consumed by volunteers every 12 hours for 4 days after undergoing bilateral lower third molar surgery with molars in similar positions using a double blinded crossover randomized design.

## Material and Methods

- Registration and Study Design

This study was performed in accordance with the Declaration of Helsinki and approved by the institutional Ethics Committee of the Bauru School of Dentistry, University of São Paulo, Brazil National Research Ethics System (CAAE number: 30317314.4.0000.5417), in accordance with resolution 466/12 of the National Council of Health / Ministry of Health, and registered with ClinicalTrials.gov (NCT02494856;https:// clinicaltrials.gov/show/ NCT02494856). All volunteers completed an Informed Consent form during screening prior to carrying out any study procedures. Sample size was determined based on previously published studies (20).

Briefly, 52 adults (≥18 yrs old) requiring bilateral lower third molars that were similarly positioned (5,21), according to the Pell and Gregory’s classification - classified by panoramic radiograph (22) were screened for participation in this study. Inclusion criteria included a lack of inflammation or infection at the extraction sites and an absence of systemic diseases that could possibly interfere with the study. Furthermore, extractions were based on orthodontic, periodontic and endodontic indications, among other dental indications (e.g. caries or periodontal pockets in the distal region of the second molar), to assess the risk of resorption of the second molar root.

Exclusion criteria included the following: history of allergy to local anesthetics or any inability to receive articaine; history of bleeding or gastrointestinal ulcers, kidney disease, asthma, or allergic sensitivity to any NSAIDs; individuals pregnant or breast-feeding; use of antidepressants within one year before the research; use of anticoagulants, diuretics and/or antibiotics within two months before surgery; and hepatic, kidney, intestinal, cardiac, pulmonary, circulatory and/or brain dysfunction (5,21). Furthermore volunteers were excluded from the study if they had adverse drug reactions (e.g. an allergic reaction to any medications) or required different doses of local anesthetic during surgery (23).

- Surgery Intervention and Assessments

**Figure 1:**
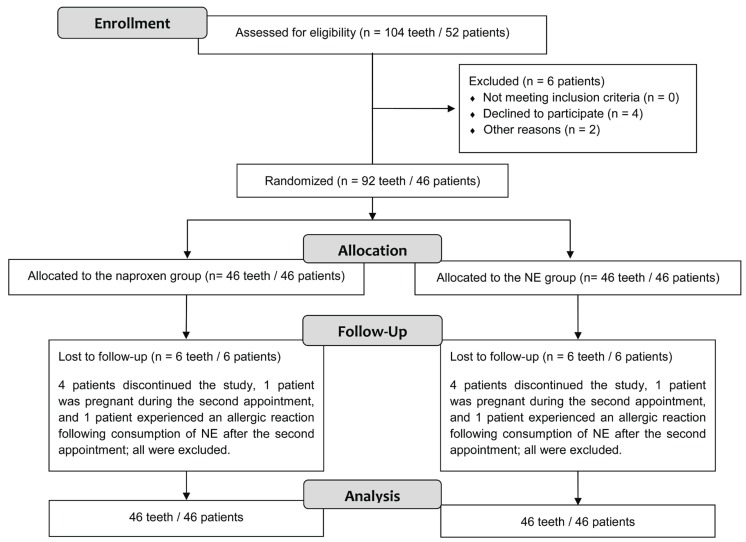
CONSORT flow diagram of the study design.

Initially, 52 volunteers who fulfilled all the criteria were screened based on their panoramic radiographs from the Bauru School of Dentistry archives, University of São Paulo (Bauru, SP, Brazil). Of these 52 patients, six were excluded from the study. Specifically, one volunteer had postoperative complications experiencing allergy, another became pregnant before the second surgery and the other four volunteers dropped out of the study. In total, there were 92 extracted third molars (46 volunteers) that were analyzed (Fig. 1).

All surgeries were performed by the same dental surgeon (GMW) using a standard protocol (5,20,21,24) and occurred from June to November 2015 at the Clinical Pharmacology and Physiology Laboratory (LAFFIC) at Bauru School of Dentistry, University of São Paulo (Bauru, SP, Brazil). All surgical procedures and the drugs provided to patients had their costs funded by São Paulo Research Foundation (FAPESP) - process number 2013 / 26467-2. The side (right or left) for the first operation and NSAID to be used (naproxen or NE) after this surgery were determined randomly (http://www.randomization.com, number: 27159). Specifically, volunteers received either a yellow or brown coded package containing the NSAID for consumption after the first surgery, and then was given the alternate package for their second surgery on the contralateral side (23,24).

Starting immediately after the first surgery, the volunteers randomly consumed either one 500 mg tablet of naproxen or one tablet with 500 mg of naproxen with a 20 mg enteric coating of esomeprazole (NE) every 12 h for 4 d. Specifically, volunteers consumed the research medication during the following times: 0, 12, 24, 36, 48, 60, 72, 84 and 96h. During the first hour, volunteers remained on the research premises and were observed while they recorded their postoperative pain using a visual analog pain scale (VAS). Volunteers were also provided with rescue medication (500 mg acetaminophen tablets) that could be consumed every 8 h to supplement the naproxen or NE medication and were instructed explicitly to maintain the use of the research medication for the entire protocol. The second surgery was performed 4 to 6 weeks after the first surgery, where the previous research medication not used was then taken.

Postoperative pain was evaluated as described in earlier studies (5,21). Briefly, volunteers evaluated postoperative pain using a VAS (0 to 100 mm) with 0 mm indicating no pain and 100 mm indicating worst possible pain. Additionally, postoperative pain was recorded by the volunteer during the following specific time points after surgery: 0, 0.25, 0.5, 0.75, 1, 1.5, 2, 3, 4, 5, 6, 7, 8, 10, 12, 18, 24, 48, 72 and 96 h (the end of surgery was considered time zero). Patients also received another VAS form without predetermined times to record the amount of pain experienced at the time when rescue medication was consumed. In addition to the forms recorded by the volunteers, a single researcher (GMW) evaluated the following parameters in the pre, intra and postoperative periods. Antibiotics were prescribed to volunteers only in cases where local oral infections were observed during follow-up visits.

- Collected data and surgical outcomes

All the parameters evaluated in this study are reported in  Table 1.

**Table 1:**
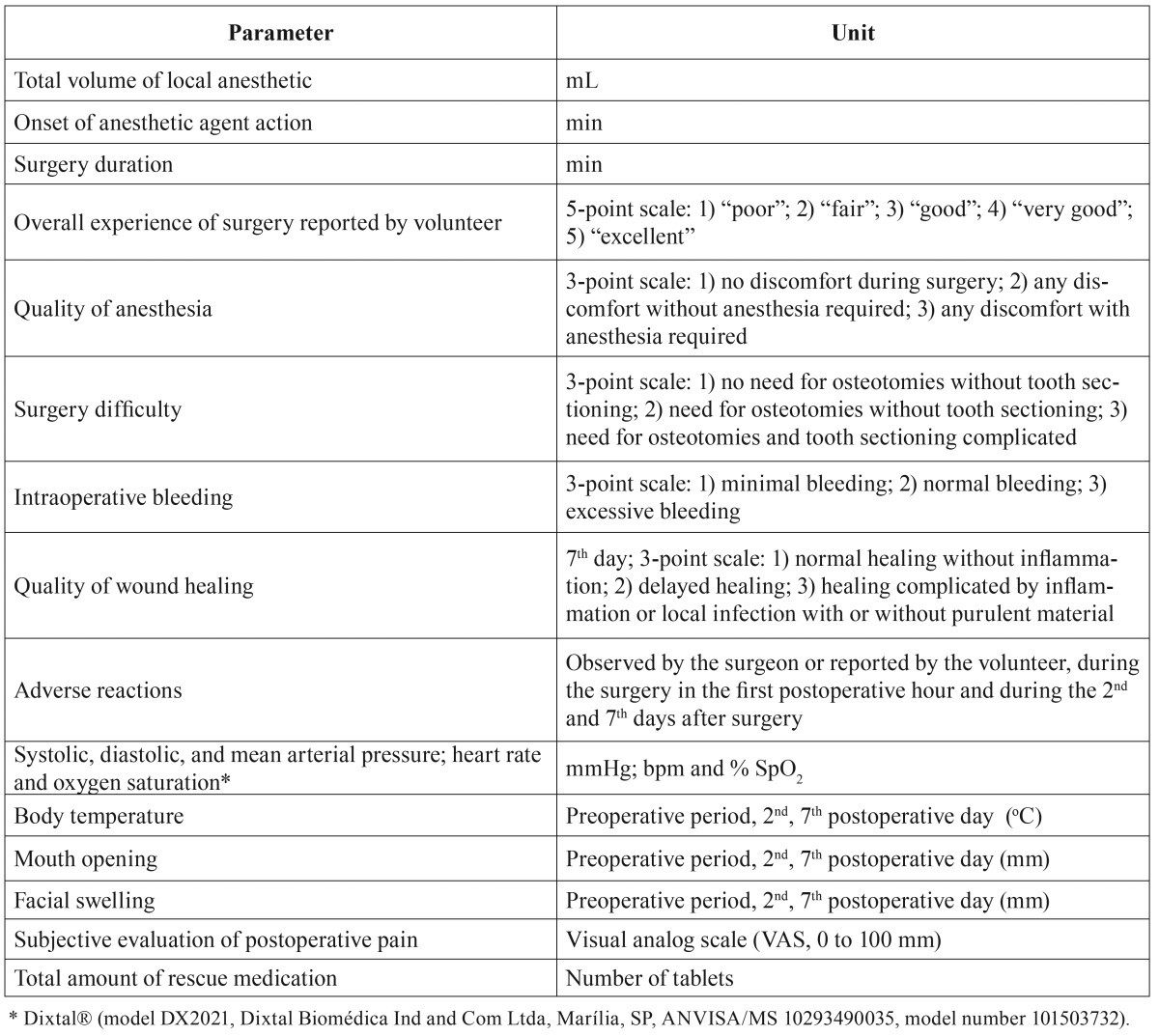
Study parameters evaluated.

- Statistical Analysis

Data were analyzed using Microsoft® Excel 2002 (version 10.6871.6870) and IBM®SPSS® statistics (version 20.0.0). Briefly, data were tested for normality using the Shapiro-Wilk test. When data were normally distributed, comparisons between the naproxen and NE groups were made using paired Student’s t-tests, and in cases where data were non-normally distributed, the Mann-Whitney U test was employed. The chi-square goodness of fit test was used to compare observed ratios with expected ratios (e.g. observed versus expected ratio of females to males). Lastly, binary data such as the absence or presence of an adverse reaction (e.g. headache) between naproxen and NE or female and male were compared using Fisher’s exact test. Statistical significance was set at 0.05. Normally distributed data are reported as a mean ± 1 standard deviation (SD) whereas non-normally distributed data are reported as median with the interquartile range [IQR].

## Results

A total of 46 volunteers (92 molars) were studied of which 34 (74%) were female aged 25 ± 6 yrs and 12 (26 %) were male aged 28 ± 7 yrs ( Table 2). The average age of all the patients was 26 yrs, with ages ranging from 18 to 44 yrs. For unknown reasons, the observed ratio of female to males (17:6) was significantly different from the expected ratio of female to males (50.43:49.57) in the age range tested (chi-square goodness of fit test, *p*< 0.001). Consequently, the naproxen and NE groups were investigated in total and separated by gender resulting in several significant findings.

**Table 2:**
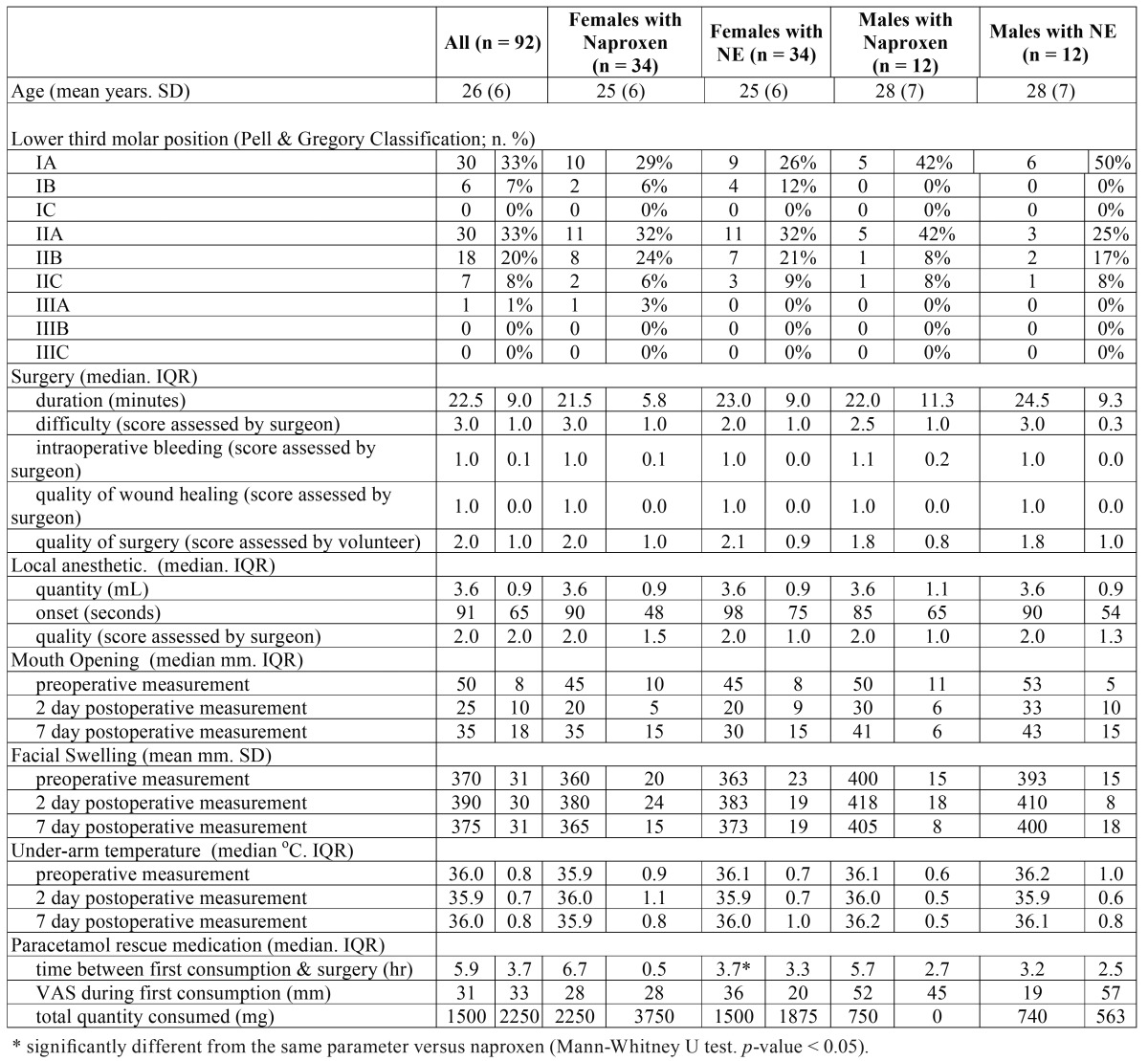
Preoperative, intraoperative and postoperative parameters.

While nine women compared to no men reported adverse reactions this observed difference was not statistically different (Fisher’s exact test, *p* = 0.105). Adverse reactions reported by women were not significantly different when they consumed naproxen compared to NE (Fisher’s exact test, *p* = 0.476). When consuming naproxen, 2 women reported headaches while a third reported nausea and diarrhea. Similarly, when consuming NE, 3 women reported headaches, 1 woman reported stomachaches, and 2 women reported vomiting with one of those women also reporting nausea. Lastly, on the 7th day after surgery, two women in the NE group had developed infections at the site of surgery and were prescribed 500 mg of amoxicillin for 8 hours for 7 days; these two patients were followed until their infection was resolved.

The time duration between the end of surgery and when female volunteers first consumed rescue medication to supplement their experimental pain medication was significantly increased when they consumed naproxen when compared to consuming NE (6.7 h [0.5] versus 3.7 h [3.3], respectively; Mann-Whitney U test, *p*= 0.03) as reported in  Table 2.

**Figure 2:**
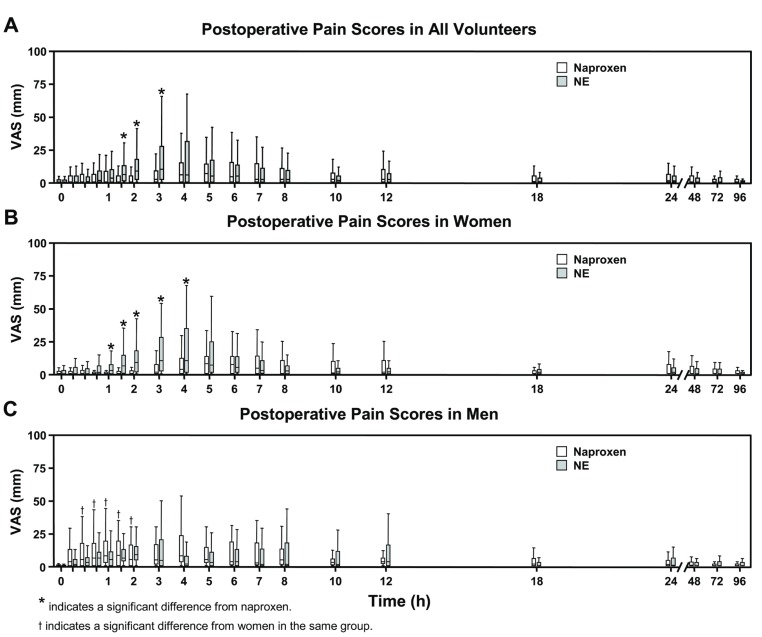
Postoperative Pain Scores with Naproxen (500 mg) or Naproxen with Esomeprazole (NE, 500/20 mg) in Volunteers. Visual analog scale (VAS) of self-reported postoperative pain scores after lower third molar surgeries assessed at 0.25, 0.5, 0.75, 1, 1.5, 2, 3, 4, 5, 6, 7, 8, 10, 12, 18, 24, 48, 72 and 96 h. Scores could range from 0 to 100 mm with larger scores indicating increased pain. Data are presented as median and IQR (n = 46). * indicates a significant difference from naproxen, Mann-Whitney U test, *p*-value ≤ 0.05. t indicates a significant difference from women in the same group, Mann-Whitney U test, *p*-value ≤ 0.05.

As indicated by the VAS (Fig. 2A), reported postoperative pain scores were no different between men when they consumed naproxen and NE. However, reported postoperative pain scores were significantly increased between women when they consumed naproxen compared to NE at time points 1, 1.5, 2, 3, and 4 h after surgery (Fig. 2B). Women who consumed naproxen alone reported significantly less postoperative pain at 0.5, 0.75, 1.0, 1.5 and 2.0 h after surgery when compared to men who consumed naproxen alone (Mann-Whitney U test, *p* < 0.05) as reported in fig. 2C.

**Figure 3:**
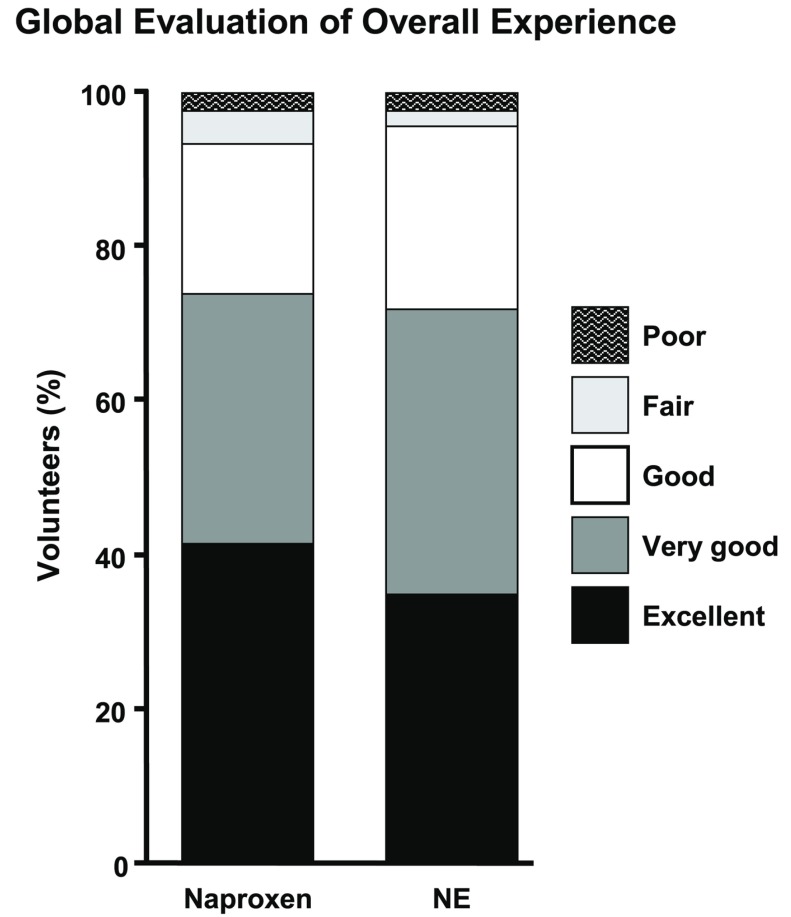
Global Evaluation of Overall Experience. Self-reported global efficacy of oral naproxen (500 mg) compared to naproxen with esomeprazole (NE, 500/20 mg). during the seventh postoperative day as assessed using a 5-level Likert scale (n = 46). The format of the Likert ratings was “excellent,” “very good,” “good,” “fair,” or “poor”. * Indicates a *p*-value ≤ 0.05, Mann-Whitney U test for the “excellent” sub-group between research medications.

All other parameters tested whether preoperative, intraoperative or postoperative were not found to be significantly different between men and women or between both genders combined when consuming naproxen or NE ( Table 2, Fig. 3). For example, mouth opening or indicators of inflammation such as swelling were no different between females or males who consumed naproxen when compared NE consumption. Specifically, increased facial swelling of men and women on the 2nd postoperative day had measurements of 105.9± 3.4% and 106.5± 7.6% respectively, when taking naproxen, compared to 104.7± 2.7% and 105.4± 3.2% respectively when taking NE ( Table 2). Similarly, on the 7th postoperative day, increased facial swelling of men and women had measurements of 101.9 ± 2.2% and 103.3± 7.8% respectively, when taking naproxen, compared to 100.7± 1.2% and 101.8± 1.7% respectively when taking NE ( Table 2).

## Discussion

To the best of the authors’ knowledge, this study is the first to investigate the clinical efficacy of naproxen versus naproxen with an enteric coating of esomeprazole (NE) using a model of acute pain immediately after surgery, followed by lower pain intensity measurements up to 96 hours after surgeries. Briefly, a double-blinded crossover randomized study design has the advantage of comparing two drugs with the same population of individuals, hopefully minimizing confounding factors introduced by comparing two populations. To ensure the integrity of the results of this crossover study all the volunteers were selected based on the bilateral positioning of their teeth with each volunteer’s molars in similar positions minimizing differences in the surgical trauma between both surgeries (20,23,24,25). In deed, each group tested had similar interoperative parameters (including hemodynamic parameters – data not shown) during surgery ( Table 2).

The manufacturer of NE (AstraZeneca; London, United Kingdoms) explicitly indicates the use of NE for chronic and not acute pain stating that “VIMOVO is not recommended for initial treatment of acute pain because the absorption of naproxen is delayed compared other naproxen-containing products”. However, according to the manufacture, “esomeprazole is rapidly absorbed with peak plasma concentration reached within, on average, 0.43 to 1.2 hours” (19) and chronic pain is clinically described as pain lasting beyond 3 or 6 months. Thus it is assumed that a dose of NE could be effective for pain lasting several days or hours i.e. acute pain. Similarly, naproxen has been reported to have a peak plasma concentration within about 1 hour (7,19).

Indeed NE was found to be effective in managing pain several hours after surgery when acute anesthesia wore off. Without any pain management, patients would report extremely grievous pains scores. Compared to other pain medications such as nimesulide, ketoprofen, ibuprofen and piroxicam, which are indicated for the treatment of acute pain and chronic pain, both naproxen and NE had comparable pain scores. Therefore, despite the indication by the manufacturer for the exclusive control of chronic pain by NE, this study found that NE was effective against acute postoperative pain, with almost all postoperative pain scores reported well below 40 mm at all time points.

It should be noted that patients consumed their first dose of naproxen or NE immediately after surgery, and that the local anesthetic used (4% articaine with 1:200,000 epinephrine) has been demonstrated to last on average 193 minutes according Gregorio *et al.* (20) (2008) or 196 minutes according Santos *et al.* (24) (2007) after administration during third molar surgeries with osteotomies. Thus, postoperative pain between 0 and approximately 3.3 hours is most likely managed by the local anesthesia while approximately between 40 minutes and 3.3 hours postoperative pain is probably being managed by a combination of local anesthesia, which is waning and the oral medication which is waxing. Therefore, even with the delayed absorption of NE reported by the manufacturer, the volunteers in this study never reported significant amounts of acute postoperative pain during any time point tested after surgery.

Jensen *et al.* (26) (2005) found that patients who had postoperative pain experiences or chronic pain with pain intensity levels of ~44 mm on a 100 mm VAS tend to describe their pain as “soft” and reported that this level of pain minimally impacts their daily activities. Overall, the reported pain score for all groups were on average well below 40 mm on the VAS throughout the entire study, which is considered low clinically; furthermore adverse reactions were minimal. Therefore, both medications should be considered effective for managing postoperative pain.

Other studies have demonstrated the clinical efficacy of different formulations (250, 400 and 500 mg) of naproxen to combat edema and pain trismus using a third molar surgery model (1-3,6,8,17,18). These formulations of naproxen in their different therapeutic doses reportedly manage postoperative pain and inflammation effectively (1,2-4,8,17,18).

Despite naproxen’s demonstrated effectiveness against inflammation and pain, it has been associated with gastrointestinal problems when used for long periods and/or at high doses (7,17,27). However, in this study adverse reactions from naproxen were minimal, and the presence of esomeprazole did not significantly reduce the number of adverse reactions to naproxen in the population of volunteers studied (NE versus naproxen). Specifically, the number of adverse reactions experienced in all groups was quite low and, in particular, men reported no adverse reactions at any time with either medication.

Other studies have found varying degrees of adverse reactions when taking naproxen after third molar surgery. For example, Varner *et al.* (6) (2009) found adverse side effects in patients (aged 22 ± 5 yrs) who consumed naproxen (550 mg) with 8 (16%) patients reporting nausea, 8 (16%) reporting headache, 4 (8%) reporting dizziness, 4 (8%) reporting vomiting and 3 (6%) diagnosed with dry socket. Whereas, Michael Hill *et al.* (8) (2006) reported that 14 out of 39 (36%) individuals (aged 25 ± 4 yrs) experienced at least one adverse reaction when they consumed naproxen (500 mg) with the most common adverse side effects being alveolitis, headaches, gingivitis, and dizziness.

NSAIDs have been shown to adversely affect older populations when compared to younger populations. However, there were no significant differences between the ages of women and men in this study, and therefore should not have been a factor in the adverse reactions to NSAIDs observed in this study.

Curiously, results from this study also suggest that naproxen alone manages acute postoperative pain in women more effectively when compared to NE, and that postoperative pain in men would be effectively managed by either medication. For the women examined in this investigation, the delay in the absorption of naproxen after the coating of esomeprazole was digested might have resulted in more postoperative pain immediately after surgery when the local anesthetic dissipated. In contrast, men did not experience a difference in postoperative pain between the two research medications, and it remains unclear why men were different from women in this respect.

Keep in mind, when women consumed NE they also on average consumed their first rescue medication (acetaminophen) much earlier then when they consumed naproxen without esomeprazole (3.7 h versus 6.7 h, respectively). Therefore, not only were women reporting more postoperative pain when taking NE when compared to naproxen, but they also consumed supplemental pain medication at a much earlier time after surgery.

It is unclear whether other studies have observed any gender differences for postoperative pain when using naproxen (or NSAIDs in general). Perhaps this study is the first to find a difference in postoperative pain between men and women when consuming naproxen.

Estrogen is also associated with increased circulating prostaglandins and prostacyclin in human and other animal experiments (15). According Amandusson & Blomqvist (10) (2013), when compared to men, women have increased pain somatization, sensitivity and intolerance for various types of pain. The authors reported that the presence of estrogen in women favors nerve transmission in spinal nociceptive circuits and supraspinal levels in the central nervous system, thus contributing to pain transmission and modulation. This may help explain increased pain sensitivity observed in women in this and other studies. Additionally, Cherney *et al.* (13) (2008) found that women were significantly sensitive to COX-2 inhibition whereas men were not. They hypothesized that women have greater baseline prostaglandin activity when compared to men and that vasodilatory prostaglandins are more effective in women versus men (13). Others using animal models have reported or implicated estrogen as a mediating factor, since estrogen increased vasodilatory prostaglandin synthesis (14-16). Taken together, COX-2 mediated mechanisms may be significantly affected by estrogen and thus by gender. Whether men reported more postoperative pain than women when consuming naproxen alone in the first two hours after surgery, due to the effects of estrogen or other sex hormones on COX-2, requires further investigation.

Currently, there are still no other randomized clinical trials evaluating the effectiveness of NE using an acute pain control model (22). NE has only been evaluated for its ability to manage chronic pain (19,28). However, its efficacy, tolerability and bioavailability have been widely tested by Choi *et al.* (27) (2015) and Angiolillo *et al.* (28) (2014).

Indirect and direct parameters investigating postoperative inflammation (e.g. mouth opening and swelling) indicate that naproxen and NE equally manage postoperative inflammation often associated with invasive oral surgery. In particular, the reduction of the mouth opening, known as trismus, during the postoperative period is reported by several authors, and it is closely linked to the level of swelling and inflammation generated by surgery. Limited mouth opening is typically associated with moderate inflammation; although it is sometimes associated with more severe trismus (29). With respect to mouth opening, this study found no significant differences between the naproxen and NE groups (in both genders) suggesting that medications are equally effective in combating postoperative trismus, a result which agrees with other studies from this laboratory (5,20,21,23,24,25). By the 7th postoperative day volunteers were able to open their mouth nearly equal to preoperative distances.

Swelling is an expected reaction after third molar extractions, with swelling being the greatest 48 hours after surgery (21). This study found no differences in swelling between volunteers when they consumed naproxen alone or with esomeprazole. There are several studies that have investigated the effectiveness of NSAIDs on inflammation originating from third molar surgeries (1-3,5,6,9). For example, Bjornsson *et al.* (17) (2003) reported that naproxen (500 mg twice daily for 3 d) reduced swelling 20.9% on the 6th day after third molar surgery although acetaminophen (1000 mg four times daily for 3 d) was more effective. In contrast, Akbulut *et al.* (1) (2014) found that naproxen was less effective against swelling when compared to diclofenac potassium. Additionally, Kara *et al.* (2) (2010) reported that naproxen sodium ineffectively reduced swelling in patients, being equivalent to placebo.

In conclusion, both naproxen (500 mg) and NE (500/20 mg) effectively manage postoperative pain and inflammation in adults after third molar surgery with pain scores rarely exceeding more than mild pain. However, in terms of postoperative pain, women benefited more when consuming naproxen alone then with esomeprazole, whereas men were no different between the two medications. Additionally, adverse side effects were minimal in volunteers and no significant differences were found between volunteers when they consumed naproxen or NE.
